# Comparison of the Fertility Outcome of Salpingotomy and Salpingectomy in Women with Tubal Pregnancy: A Systematic Review and Meta-Analysis

**DOI:** 10.1371/journal.pone.0152343

**Published:** 2016-03-25

**Authors:** Xiaolin Cheng, Xiaoyu Tian, Zhen Yan, Mengmeng Jia, Jie Deng, Ying Wang, Dongmei Fan

**Affiliations:** Department of Obstetrics and Gynecology, The First Affiliated Hospital of Henan University of Science and Technology, Luoyang, Henan Province, China; University of Rochester, UNITED STATES

## Abstract

**Objective:**

To compare the natural fertility outcomes of salpingotomy and salpingectomy among women treated for tubal pregnancy.

**Methods:**

An online database search including PubMed, Embase, CENTRAL and Web of Science was performed to identify studies comparing salpingotomy and salpingectomy to treat women with tubal pregnancy. The search included papers published after the databases were established until May 2015. Two reviewers independently screened literature according to the inclusion and exclusion criteria and then extracted data and assessed the methodological quality of all of the included studies. The meta-analysis was conducted using RevMan 5.3 software. The registration number is CRD42015017545 in PROSPERO.

**Results:**

Two randomized controlled trials (RCTs) and eight cohort studies, including a total of 1,229 patients, were znalyzed. The meta-analysis of the RCT subgroup indicated that there was no statistically significant difference in IUP rates (RR = 1.04, 95% CI = 0.89–1.21, P = 0.61) nor the repeat ectopic pregnancy (REP) rate (RR = 1.30, 95% CI = 0.72–2.38, P = 0.39) between the salpingotomy and salpingectomy group. In contrast, the cohort study subgroup analysis revealed that the IUP rate was higher in the salpingotomy group compared with the salpingectomy group (RR = 1.24, 95% CI = 1.08–1.42, P = 0.002); Salpingotomy also increased the risk of REP rate (RR = 2.27, 95% CI = 1.12–4.58, P = 0.02). The persistent ectopic pregnancy (PEP) occurred more frequently in the salpingotomy group than the salpingectomy group (RR = 11.61, 95% CI = 3.17–42.46, P = 0.0002). An IUP would be more likely to occur after salpingotomy than salpingectomy when the follow-up time was more than 36 months (RR = 1.16, 95% CI = 1.02–1.32, P = 0.03). The IUP rate (RR = 1.13, 95% CI = 1.01–1.26, P = 0.03), and the REP rate (RR = 1.62, 95% CI = 1.02–2.56, P = 0.04) was higher after salpingotomy than salpingectomy among patients from Europe compared with those from America.

**Conclusions:**

Based on the available evidence, we believe that for patients with a healthy contralateral tube operated for tubal pregnancy, the subsequent fertility after salpingectomy and salpingotomy are similar in the long term. The fertility prospects will not be improved via salpingotomy compared with salpingectomy.

## Introduction

Ectopic pregnancy is a common, gynecologic, acute abdominal condition that remains life threatening; in fact, it is the leading cause of maternal death in early pregnancy [1]. To date, the incidence of ectopic pregnancy has increased from 0.5% in 1970 to 2% [2]. Approximately 98% of ectopic pregnancies occur in the fallopian tube [3]; however, blastocysts can also implant in the ovary, the cornual region, a hysterectomy scar, the abdomen, or the cervix. The treatment options for tubal pregnancy include expectant management, medical treatment and surgery (conducted via laparotomy or laparoscopy). More than three-quarters of women who experience ectopic pregnancy should to be treated surgically [4]. Currently, laparoscopic surgery is the most preferred treatment option.There are two types of surgical procedure for tubal pregnancy: radical (salpingectomy) and conservative (typically salpingotomy). Salpingectomy was the standard procedure for ectopic pregnancy [5] until 1978, when laparoscopic salpingotomy was first reported by Bruhat et al.[6].In clinical practice, the choice of salpingotomy versus salpingectomy depends on many factors, including patient age, tube condition, serum human chorionic gonadotropin (hCG) levels, and patient's future fertility desire. Ectopic pregnancy is a common and serious health problem among nubile women; thus, fertility outcomes after surgery have drawn substantial attention. Theoretically, the preservation of the tube via salpingotomy should partially increase the probability of intrauterine pregnancy (IUP). Conservative management has generally been considered as the first-line treatment for patients with ectopic pregnancies who desire to have children in the future, especially those with a damaged bilateral tube. Moreover, salpingectomy has been adopted for women with a ruptured tubal pregnancy or those with uncontrolled tubal bleeding and a severely damaged tube. In some cases, the decision is primarily made by the patient.

The decision of whether to preserve or remove the tube when treating women with tubal pregnancy has been debated for many years, and controversy remains. Previous trials have resulted in different conclusions [7–10]. Whether salpingotomy improves postoperative fertility outcomes compared with salpingectomy remains unclear. Thus, a meta-analysis is needed to assess fertility outcomes after salpingectomy versus salpingotomy.

## Materials and Methods

The protocol of this review was registered in PROSPERO, and the registration number is CRD42015017545 (http://www.crd.york.ac.uk/PROSPERO/). The protocol and the PRISMA checklist have been uploaded as supporting information. See S1 PRISMA Checklist and S1 Protocol.

### Inclusion criteria

The inclusion criteria for this research were as follows. 1) All randomized controlled trials (RCTs) and cohort studies (prospective or retrospective) comparing the fertility outcomes of conservative surgery and salpingectomy to treat women with tubal pregnancy were included. 2) Because Dubuisson et al. [11] reported that approximately 93% of all spontaneous pregnancies after ectopic pregnancy surgery occur in the 18 months following the procedure, and Bouyer et al. [12] reported that spontaneous pregnancies following ectopic pregnancy often occurred 18–24 months after treatment, we therefore defined the follow-up time of the current review as 2 years or longer. 3) All included studies had their diagnoses confirmed via laparoscopy or pathology. 4) All included participants had healthy contralateral tubes and attempted to become pregnant after surgery. 5) All salpingotomies were performed via laparoscopy or laparotomy, regardless of whether the incision was surgically closed.

### Exclusion criteria

Studies were excluded from this research if 1) the ectopic pregnancy occurred at a non-tubal site (e.g., cornual or cervical pregnancies); 2) the patients had infertility diseases or infertility factors such as a previous tubal surgery; 3) the patients had complete tubal abortions or pregnancies via in-vitro fertilization (IVF) or embryo transfer; 4) the patients with fimbrial tubal pregnancy treated via “milking” (i.e., squeezing the products of conception through the fimbria); 5) they had a follow-up time of less than 2 years; and 6) they did not enable data extraction. We did not account for laparotomy versus laparoscopy because this difference in approach is not associated with a rate of subsequent IUPs or repeat ectopic pregnancies (REPs) [5,13,14].

### Types of outcome measures

The primary outcome was postoperative intrauterine pregnancy (IUP) by natural conception. An IUP was defined as an ongoing pregnancy confirmed via ultrasound or the birth of a child, including IUP abortion and IUP delivery. The secondary outcome was postoperative repeat ectopic pregnancy (PEP) and persistent ectopic pregnancy (PEP). PEP refers to the recurrence of ectopic pregnancy during the follow-up period. All recurrences were included, regardless of site.

### Search strategy

Electronic databases including PubMed, the Science Citation Index (SCI), Cochrane Central, EMBASE, CINAHL, and Web of Science were searched. Both the medical subject heading (MeSH) and text-word searches were employed using the following terms: “ectopic pregnancy”, “tubal ectopic pregnancy”, “tubal pregnancy”, “salpingotomy”, “conservative surgery”, “radical surgery”, “salpingectomy”, and “tubal resection”. All of the references included in this article were also searched. The language of the literature was limited to English.

### Data collection

Two well-trained and qualified reviewers independently selected all of the literature based on the inclusion and exclusion criteria. Information regarding the literature, including the authors’ names, institutions, or journals, was extracted by the reviewers in the selection process. Any disagreement was initially resolved via discussion; if a consensus could not be reached, then a third reviewer was consulted. If two or more reports included the same participants, then only the most recent report was included in the meta-analysis. The reviewers independently extracted the relevant data, the methodological details and the baseline characteristics from the eligible literature using a standardized form. When analyzing the IUP and REP, only the data from patients with no fertility-reducing factors were extracted.

### Methodological quality assessment of the included studies

All included RCTs were evaluated using Cochrane’s tool for the risk of bias assessment [15]. This tool consists of the following seven items: (1) random sequence generation; (2) allocation concealment; (3) blinding of participants and personnel; (4) blinding of outcome assessment; (5) incomplete outcome data; (6) selective reporting; and (7) other sources of bias. Each item had three outcomes, which included the following: low bias risk, high bias risk and “unclear” (for cases in which the literature did not provide sufficient information or uncertain information needed to assess bias). The methodological quality of the included cohort studies was assessed using the Newcastle-Ottawa Scale (NOS) [16]. Its contents include the following three aspects: selection, comparability and exposure. Each aspect consists of several evaluation items, which were awarded a “☆” when the requirements were met. The total score was 9, and studies with a score of ≥7 were identified as relatively high quality.

### Statistical analyses

Statistical analyses were performed using RevMan version 5.3, and risk ratios (RRs) and 95% confidence intervals (CIs) with a level of α = 0.05 were used as the effective value to measure the outcomes. The chi-square test and Higgins’ I^2^ statistic were used to test study heterogeneity. When P≥0.1 and I^2^≤50%, the included studies were considered to have little heterogeneity, and a fixed-effects model was used. A random-effects model was used when P<0.1 and I^2^>50%, and the included studies were considered as having substantial heterogeneity. Subgroup and sensibility analyses were used to determine the cause of the heterogeneity. If unsolvable heterogeneity was encountered, then a descriptive analysis was conducted. A visual assessment of the funnel plots and quantitative assessments such as Begg’s rank correlation method and Egger’s weighted regression method were used to evaluate the potential publication bias of the included studies. These analyses were conducted using STATA 12.0. P-values less than 0.05 based on Begg’s or Egger’s tests or asymmetric funnel plots indicated a potential publication bias.

## Results

### Screening results

**Fig 1:**
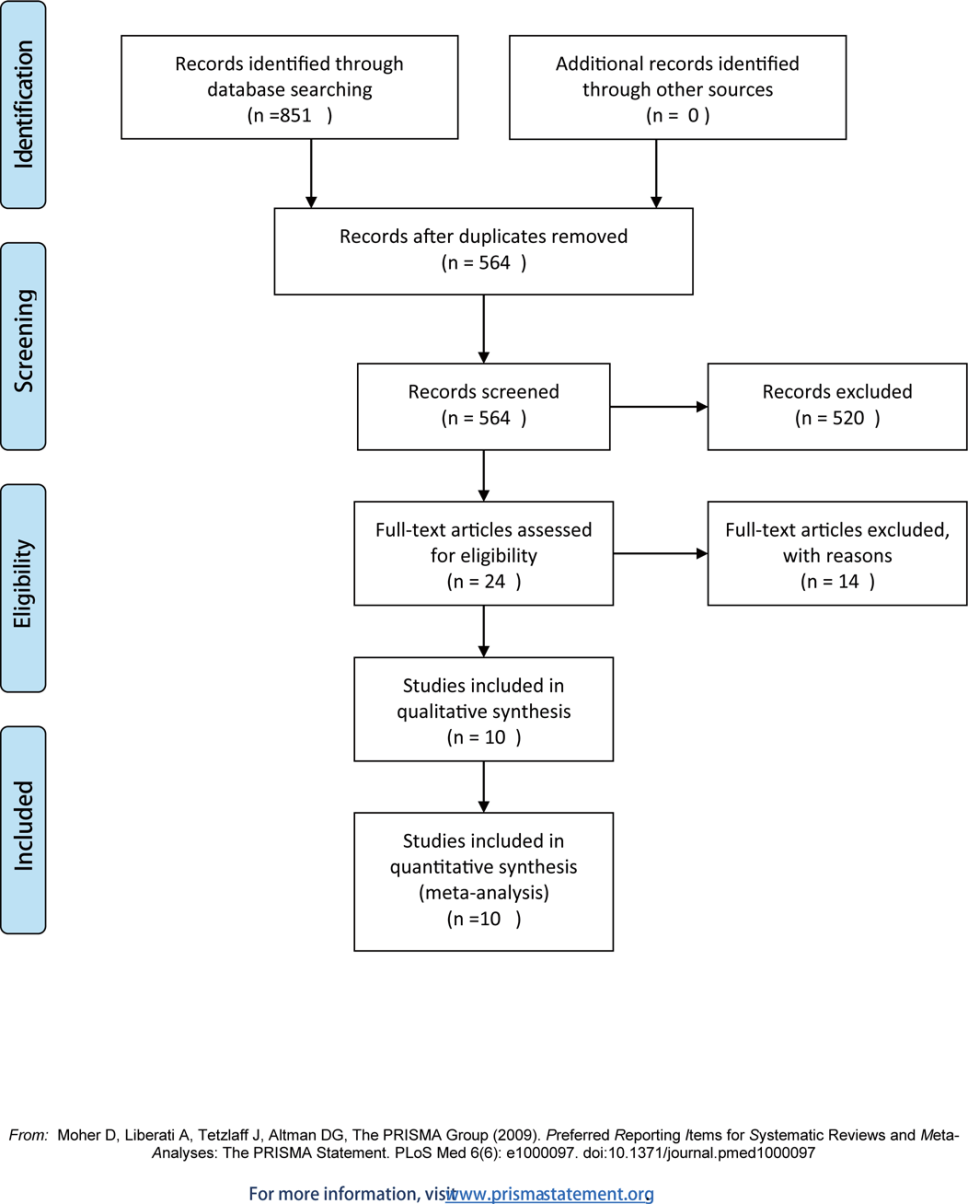
Flow diagram of the included studies.

As shown in **Fig 1**, 851 articles were initially identified using a comprehensive search. A total of 564 studies remained after the duplicate results were removed. Of these studies, 520 were deemed as irrelevant and did not accord with the inclusion criteria; thus, they were excluded after reading the abstracts. A total of 24 potentially eligible articles were reviewed by analyzing the full text. Based on this analysis, 10 published studies were included [7, 17–25] in this meta-analysis.

### Study characteristics and quality assessment

The selected studies were conducted across 7 countries and published between 1993 and 2014; however, only two [7, 17] of the 10 studies were RCTs. The remaining eight articles [18–25] were cohort studies. A total of 1,229 participants were included, 666 in the salpingotomy group and 563 in the salpingectomy group. The major characteristics of the included studies are shown in **Table 1**.

**Table 1:** Main characteristics of all of the included studies.

Study ID	Country	Study design	Age (year)	Sample size	operation style	Outcome	Follow-up (month)
				N(C/R)^①^			
Fernandez, H. 2013	France	RCT	A:31.25 B:29.28	129(63/66)	laparoscopy	IUP, REP	24 m
Mol, F. 2014	multicenter	RCT	≥18	446(215/231)	laparoscopy	IU, PEP, REP	36 m
Turan, V.2011	Izmir, Turkey	Cohort study	18–28	99(37/62)	laparoscopy laparotomy	IUP, REP	24 m
Ozler, Ali 2012	Diyarbakır, Turkey	Cohort study	28–30	76(28/48)	laparoscopy laparotomy	IUP, REP	24 m
Langebrekke, A. 1993	Norway	Cohort study	unclear	150(74/76)	laparoscopy	IUP, REP	37 m
Silva, P. D. 1993	USA	Cohort study	28.7±4.8	86(60/26)	Laparoscopy	IUP	>24 m
Mol, B. W. J. 1998	Holland	Cohort study	A:31.4 B:30.1	96(46/50)	laparoscopy laparotomy	IUP, PEP	36 m
Becker, S. 2011	Germany	Cohort study	30	85(77/8)	Laparoscopy	IUP, REP, PEP	60 m
Dela Cruz, A. 1997	Canada	Cohort study	A:28±0.6 B:27.7±0.9	90(34/56)	Laparoscopy	IUP, REP, PEP	36 m
Lermann, J 2014	Germany	Cohort study	A:33.7 B:30.8	76(63/13)	laparoscopy	IUP, REP	≥24 m

RCT = Randomized controlled study; N(C/R): N: total number; C: number in the salpingotomy group; R: number in the salpingotomy group; A: age in the salpingectomy group B: age in the salpingotomy group; IUP: postoperative intrauterine pregnancy; REP: repeat ectopic pregnancy; PEP: persistent ectopic pregnancy

The two RCTs used a computer-generated random sequence, and a random allocation was generated and concealed; thus, the risk for sequence generation was determined to be low. The patients assigned to the types of surgery were not blinded to group allocation in the two studies; however, the researchers who collected the data were blinded to the assigned intervention. We decided that the outcome measurement was unlikely to be influenced by the lack of blinding; thus, all studies were determined to have low attrition bias risk.

For the dropout patients, the TTP was analyzed and reported in both studies. All studies reported the primary outcome, and they were identified as low risk for selective reporting. **Table 2**shows the methodological quality of the two RCTs. The results of the quality assessment of the cohort studies are shown in **Table 3,** and the scores ranged from 6 to 8. All of studies were identified as relatively high quality.

**Table 2:** Quality assessment of the RCTs included in the meta-analysis.

Study ID	Selection bias		Performance bias	Detection bias	Attrition bias	Reporting bias
	Random sequence generation	Allocation concealment	Blinding of participants and personnel	Blinding of outcome	Incomplete outcome data	Selective reporting
Mol, F2014	low	low	low	low	low	low
Fernandez 2013	low	low	low	unclear	low	low

**Table 3:** Quality assessment of the cohort studies included in the meta-analysis.

Study ID	selection				comparability		Outcome			Total score
	1	2	3	4	1	2	1	2	3	
Turan, V.2011	☆	☆	☆	☆	☆	-	☆	☆	☆	8
Ozler, Ali 2012	☆	☆	☆	☆	☆	-	☆	☆	☆	8
Langebrekke, A. 1993	☆	☆	☆	☆	☆	-	-	☆	☆	7
Silva, P. D. 1993	☆	☆	☆	☆	☆	☆	☆	☆	-	8
Mol, B. W. J. 1998	☆	☆	☆	☆	☆	☆	☆	☆	-	8
Becker, S. 2011	☆	☆	☆	☆	☆	-	☆	☆	-	7
Dela Cruz, A. 1997	☆	☆	☆	☆	☆	☆	☆	☆	-	8
Lermann 2014	☆	☆	☆	☆	☆	-	☆	☆	☆	8

### IUP via natural conception after salpingotomy versus salpingectomy

All of the included studies (2 RCTs and 8 cohort studies) reported IUPs post-surgery. The subgroup analyses were conducted based on the RCTs and cohort studies, and their results were divided into two groups as follows.

#### RCT subgroup analysis

**Fig 2:**
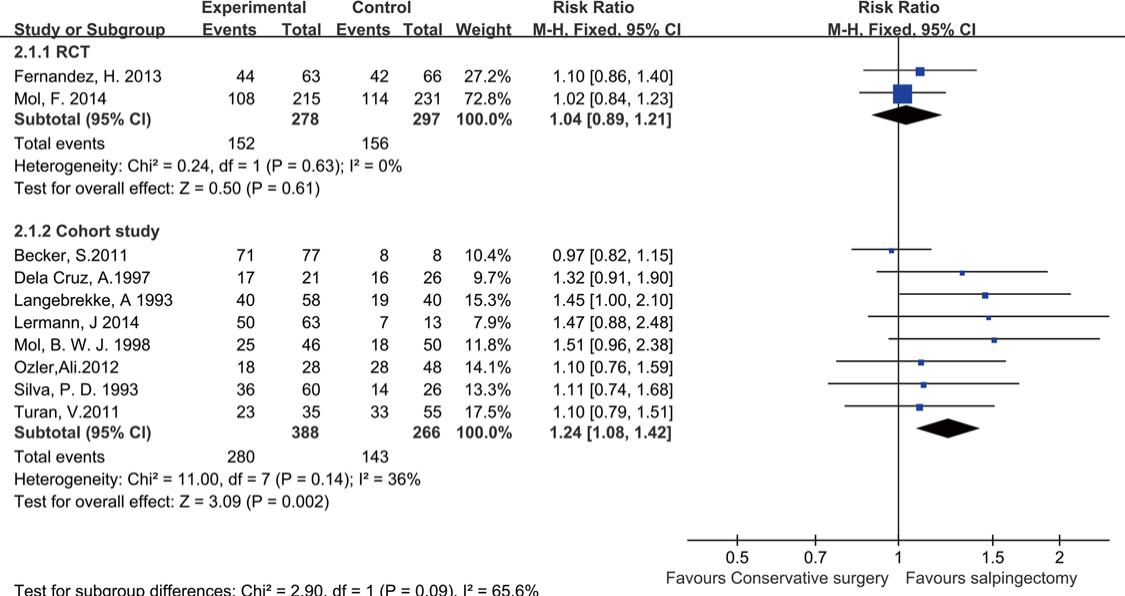
Forest plots of the meta-analysis results comparing the IUP rate after salpingotomy or salpingectomy.

There were two studies [7, 17] in the RCT subgroup. No significant heterogeneity was identified between these studies (P = 0.63, I^2^ = 0%); thus, a fixed-effects model was used to pool the outcomes in this subgroup analysis. The results showed that there was no statistically significant difference in IUP rates between the salpingotomy and salpingectomy group (RR = 1.04, 95% CI = 0.89–1.21, P = 0.61) **(Fig 2)**.

#### Cohort study subgroup analysis

Eight studies [18–25] comprised the cohort study subgroup. No significant heterogeneity (P = 0.14, I^2^ = 36%) existed, and a fixed-effects model was used for this subgroup analysis. The results demonstrated that the IUP rate was higher in the salpingotomy group than in the salpingectomy group (RR = 1.24, 95% CI = 1.08–1.42, P = 0.002) **(Fig 2)**.

According to previous studies [14,26–28], many other factors, such as age, size of the ectopic mass, the level of the serum hCG, could influence fertility following a tubal pregnancy. However, subgroup analyses based on these confounds were not possible because of a lack of data. Thus, a subgroup analysis based on several selected factors (e.g., follow-up time and ethnic background) that might influence fertility outcomes was conducted.

#### Follow-up subgroup analysis

**Fig 3:**
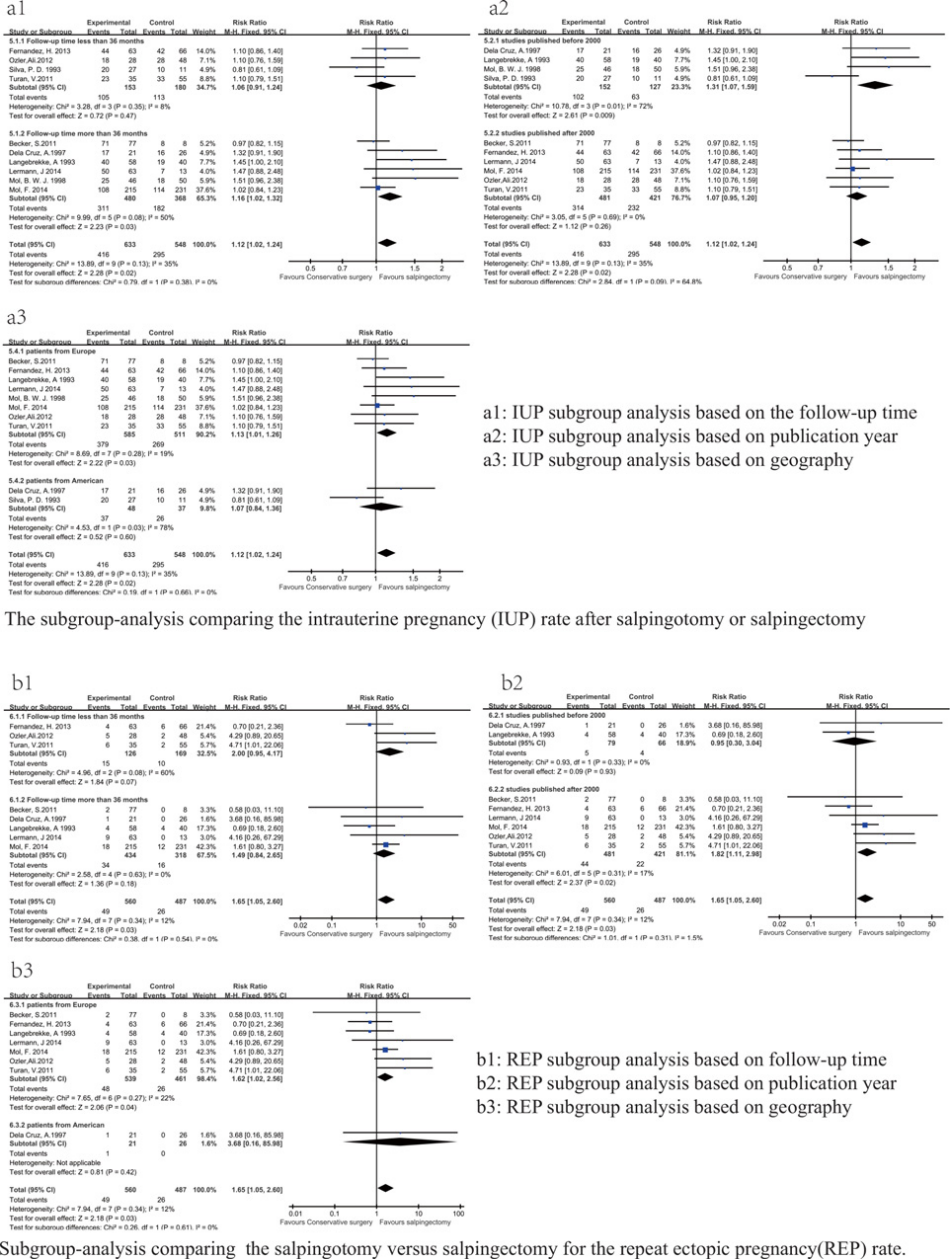
Forest plots of the subgroup-analysis based on selected items.

Four studies [17–19,21] were included in the “follow-up period of less than 36 months” subgroup, and 6 studies [7,20,22–25] were included in the “follow-up period of more than 36 months” subgroup; neither subgroup showed significant heterogeneity (P = 0.13, I^2^ = 35%). We found that an IUP was more likely to occur following salpingotomy compared with salpingectomy when the follow-up period was more than 36 months, the pooled HR calculated by the random effect model was 1.16 (95% CI: 1.02–1.32, P = 0.03; **Fig 3A1**)

#### Publication time subgroup analysis

Of the 10 included studies, 4 [20–22,24] were published in the 1990s. Laparoscopy was developed in the early 1990s [14]. During that time, laparoscopy underwent an experimental period. The remaining 6 studies [7,17–19,23,25] were published after 2000 at which point laparoscopy has been widely applied. Both the technology and equipment needed for this procedure have reached their maturity, which might introduce discrepancies in reproductive outcomes. Thus, we conducted a subgroup analysis based on when the study was published. A higher IUP rate after salpingotomy was identified in the studies published before 2000 (RR = 1.31, 95% CI = 1.07–1.59, P = 0.009; **Fig 3A2)**. In contrast, the studies published after 2000 showed no significant difference in the IUP rate between the salpingotomy and salpingectomy groups (RR = 1.07, 95% CI = 0.95–1.20, P = 0.26; **Fig 3A2)**.

#### Geographic subgroup analysis

Two studies [21,24] were included in the “patients from America” subgroup, and 8 studies [7,17–20,22,23,25] were included in the “patients from Europe” subgroup. In this subgroup analysis, the IUP rate after salpingotomy was higher among patients from Europe (RR = 1.13, 95% CI = 1.01–1.26, P = 0.03; **Fig 3A3)**.

### Repeat tubal ectopic pregnancy after salpingotomy versus salpingectomy

**Fig 4:**
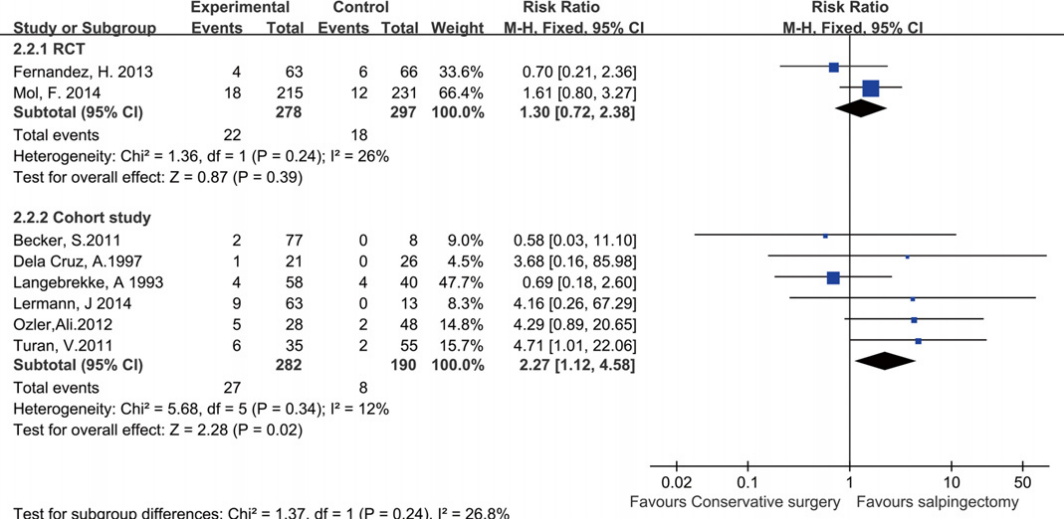
Forest plots of the meta-analysis results comparing the REP rate after salpingotomy or salpingectomy.

Eight studies (2 RCTs and 6 cohort studies) reported ectopic pregnancies after surgery. Subgroup analyses were conducted as previously described. The results of subgroup-analyses based on the RCTs and cohort studies indicated that the REP rate did not significantly differ between the salpingotomy and salpingectomy groups (RR = 1.30, 95% CI = 0.72–2.38, P = 0.39) in the RCT subgroup (P = 0.24, I^2^ = 26%), whereas in the cohort studies subgroup, It was noticed that the REP rate was higher in the salpingotomy group compared with the salpingectomy group (RR = 2.27, 95% CI = 1.12–4.58, P = 0.02; **Fig 4)**.

Moreover, The subgroup analysis based on follow-up time indicated that the REP rate was not related to the follow-up time. The REP rates for the salpingotomy and salpingectomy groups did not significantly differ in either the “follow-up time of less than 36 months” subgroup (RR = 2.00, 95% CI = 0.95–4.17, P = 0.07) or the “follow-up time of more than 36 months” subgroup (RR = 1.49, 95% CI = 0.84–2.65, P = 0.18; **Fig 3B1)**.

The publication time subgroup analysis identified an higher REP rate after salpingotomy among the studies published after 2000 (RR = 1.82, 95% CI = 1.11–2.98, P = 0.02; **Fig 3B2)**.

The subgroup analysis based on geographic indicated that the REP was more likely to occur among Americans (RR = 1.62, 95% CI = 1.02–2.56, P = 0.04; **Fig 3B3**).

### PEP after salpingotomy versus salpingectomy

**Fig 5:**
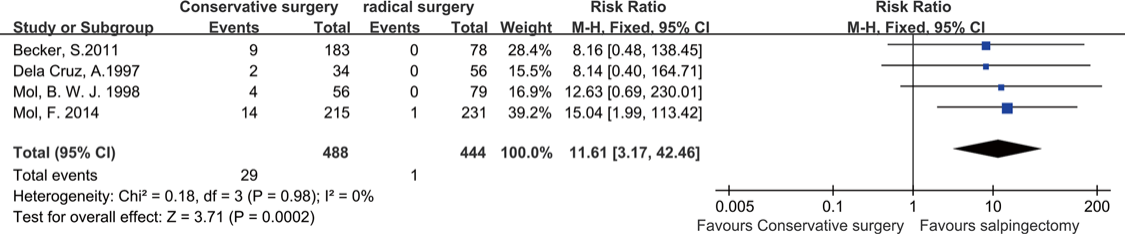
Forest plots of the meta-analysis results comparing the PEP rate after salpingotomy or salpingectomy.

Four studies reported incidences of persistent ectopic pregnancy. No significant heterogeneity (P = 0.98, I^2^ = 0%) existed, so a fixed mode was used. The results showed that that persistent ectopic pregnancies occurred more frequently in the salpingotomy group compared with the salpingectomy group (RR = 11.61, 95% CI = 3.17–42.46, P = 0.0002 **Fig 5)**.

### Publication bias and sensitivity analysis

**Fig 6:**
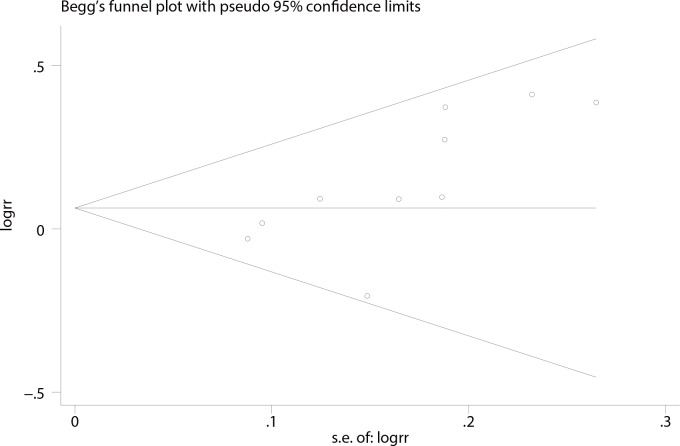
Funnel plots of the studies included in the IUP meta-analysis of via natural conception after treatment.

The publication bias of the included studies was evaluated using funnel plots as well as Begg’s and Egger’s tests. No asymmetry of the funnel plot was observed, and in the Begg’s test the P value was 0.01, suggesting that publication bias was not present in our meta-analysis **(Fig 6)**.

**Fig 7:**
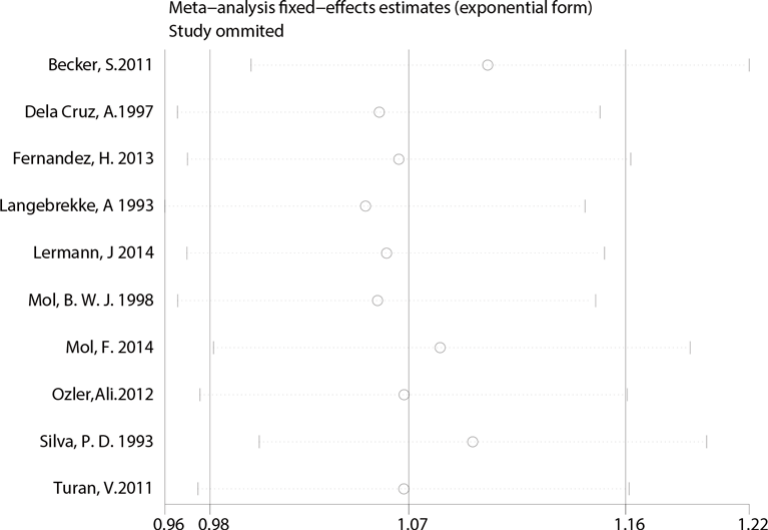
Sensitivity analysis of studies comparing IUP.

**Fig 8:**
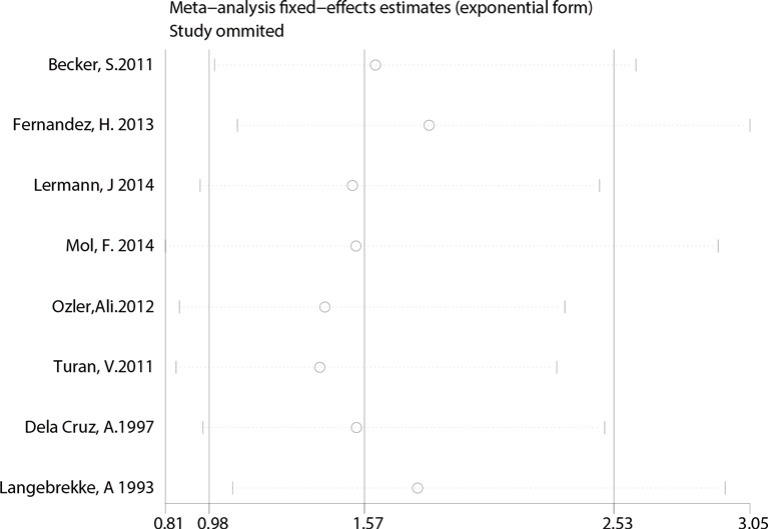
Sensitivity analysis of studies comparing REP.

Finally, A sensitivity analysis was performed to test for possible bias. Each study included in our meta-analysis was omitted from each round. The pooled RRs of IUP and REP were not significantly altered by the removal of any study, indicating that our results were statistically robust (**Fig 7 and Fig 8)**.

## Discussion

Because of ethical limitations, most published studies comparing salpingectomy and salpingotomy to treat ectopic pregnancy are retrospective and observational. In the absence of high-level evidence, we included both RCTs and cohort studies.

However, the results of the subgroup analysis between the RCTs and the cohort studies indicated different findings. The cohort studies indicated higher IUP and REP rates in the salpingotomy group, whereas the two RCTs did not indicate significant differences in the subsequent IUP or REP rates between the salpingotomy and salpingectomy groups.

These differences are highly influenced by the design features of the cohort studies. In most of the included cohort studies, the decision between salpingotomy and salpingectomy depended on the state of the fallopian tube. The patients were not randomly allocated to different treatments, which might have introduced a selection bias. In order to minimize heterogeneity, we tried to control for potential confounding factors to ensure that no significant differences existed in the basic patient characteristics when we included the cohort studies; however, a selection bias inevitably exists.

Furthermore, we find that the results of the cohort and RCT subgroups were consistent when the studies by Langebrekke.1993 and Mol, B. 1998 were excluded. In an in-depth analysis of Langebrekke.1993, it was noticed that the patients who received salpingectomies (52% [40/76]) had less desire for fertility than those who chose the conservative surgery (78% [58/74]). We speculate that this difference might cause a lower fertility rate in the radical surgery group compared with the conservative surgery group.

In further analysis of the study by Mol, B.in 1998, although the study showed higher 3-year cumulative pregnancy rate in the conservative surgery group than the control group (salpingectomy) (P <0.001, log-rank test), however, this author reported that pregnancy continued to occur in the salpingectomy group at the end of the follow-up period. Therefore, the authors also speculated that no significant difference in the cumulative pregnancy rate exists between the two groups when the follow-up time is prolonged. This finding is also supported by Ory et al [28] who demonstrated that no difference existed in fertility outcomes when 88 patients treated with linear salpingotomy or salpingectomy were followed up for 12 years.

Similar findings were identified in our subgroup analysis based on follow-up time; we found that an IUP was more likely to occur after salpingotomy than salpingectomy when the follow-up time was greater than 36 months. The follow-up times conducted by those two RCT studies were less than 36 months, but 75% of the cohort studies had a greater than 36 months follow-up time. This result suggested that the effect of salpingectomy could be better on IUP incidence than salpingotomy in the long term.

De Bennetot et al. [27] analyzed 1,064 patients with ectopic pregnancy in a prospective, population based-study. The crude 2-year cumulative rate of IUP was lower after a radical treatment (67%) compared with a conservative treatment (76%). However, a univariate analysis indicated that the pregnancy rate in the radical surgery group was lower than that in the conservative surgery group. A multivariate analysis did not indicate significant differences in the fertility rate between the two methods, because this analysis adjusted for confounds and removed interference factors. Similarly, Bangsgaard et al. [10] found that no significant difference existed in the postoperative recurrence rate of ectopic pregnancy after removing the interference of the confounding factors (88% vs. 66%; log rank P<0.05). Thus, we hypothesized that no differences exist in the postoperative IUP rates between the two surgical methods, regardless of study type, after removing potential confounds.

The subgroup analyses also found that the IUP and REP rates were higher after salpingotomy than salpingectomy among patients from Europe compared with those from America. We speculate that this finding is related to the effects of region. The concept of pregnancy differs across ethnic and linguistic groups. However, none of the 10 reviewed articles included data from Asian or African countries. Thus, the studies were only divided into those that sampled “patients from America” and those that sampled “patients from Europe”.

The studies published before 2000 identified a higher IUP rate after salpingotomy. This finding was primarily caused by the differences in the surgical techniques and the laparoscopic equipment used as previously described. Needless to say, the quality of the previous surgery is related to the REP rate. But a higher REP rate after salpingotomy was observed in studies published after 2000, this results are contrary to expectations.

A skilled and experienced physician can minimize the chance of pregnancy villus residue and reduce the damage to the fallopian tube caused by the surgical instruments. We failed to collect enough information about the comparison of the persistent ectopic pregnancy rate, which is closely related to the quality of salpingotomy and can reflect the experience of the surgeon.

The results of the RCTS showed that preservation of the tube via salpingotomy did not provide improved fertility. This lack of an effect is largely because the transport function of the tube is damaged by the mechanical damage, and the tube is burned by bipolar electric coagulation during the course of the operation. Although the anatomical structure of the tube is preserved, the preserved tube might not be available. In addition, as a result of the operation-induced wound, secretion of cytokines, prostaglandin (PG) and leukocyte chemotactic factors by the tubal tissues would exert a negative effect on the reflux in the capillaries and lymphatic system, leading to postoperative tubal adhesion and hydrosalpinx. Consequently, future pregnancies would be affected.

It is unclear whether pregnancies occurred through the preserved fallopian tube. As a study [29] reported, the status of the contralateral tube might be an important factor that could influence a woman's fertility after a tubal pregnancy. Specifically, if the function of the contralateral tube is normal, more than 80% of the patients could achieve an average fertility rate.

Oelsner et al.[30] reported that whether the tube regained its normal function after conservative surgery can only be studied in patients with a single tube. Twenty-two women with a tubal pregnancy in a single tube who underwent conservative microsurgical treatment were studied two years post-surgery. Approximately 76% of the patients who hoped to become pregnant after surgery did conceive. The IUP rate was 47.6%; however, the sample size was small, and three of the patients were treated by milking (squeezing the products of conception through the fimbria). Hence, further research is needed to determine whether the restoration of tubal function following salpingotomy is achieved.

One disadvantage of salpingotomy is the increased risk of PEPs. A previous study indicated that PEPs after salpingotomy occurred in 5% to 20% of cases via laparoscopic surgery and 3% to 5% of cases via laparotomy [31]. In our meta-analysis it was also showed that the persistent ectopic pregnancy rate was higher in the salpingotomy group. The advantage of salpingotomy is its preservation of the tube for potential future fertility. Moreover, the preservation of the tube helps to maintain ovary function. Salpingectomy might decease the ovarian function [32–33].Most recent studies on this subject have focused only on fertility outcomes after surgery. Furthermore, the effects of salpingectomy on ovarian function have not been taken into account. The blood supply of the ovary originates from both the ovarian artery and the ovarian branch of the uterine artery. These branches of arteries anastomose into nets in the mesosalpinx. Blood circulation is easily damaged during salpingectomy, and the destruction of the ovarian blood supply can lead to ovarian dysfunction [34]; however, no information was provided regarding the effect that the two procedures have on ovarian function. The effects of salpingectomy on ovarian function should also be considered when determining the surgical regimen.

## Limitations

The limitations of our meta-analysis are as follows. (1) The small number of RCTs and their small sample sizes leads to insufficient statistical power, which affects the stability of the results. (2) We only included English-language literature from European and North American countries; data from African and Asian countries were not included. (3) As a result of the lack of data, we are unable to conduct a subgroup meta-analysis based on other factors such as age. Previous studies have reported that age is an important factor that affects fertility outcomes.

## Conclusions

According to the available evidence, we believe that for patients with a healthy contralateral tube operated for tubal pregnancy, the subsequent fertility after salpingectomy and salpingotomy are similar in the long term. Additional multi-center, high quality RCTs with large samples are required for further verification. The fertility prospects will not be improved via salpingotomy compared with salpingectomy, moreover, salpingotomy can be complicated by persistent ectopic pregnancy. We suggest that salpingectomy should be chosen for women with a tubal pregnancy if the contraldteral tube appears healthy. In clinical practice, both the subsequent fertility and ovarian function should be considered when making operative procedure decisions.

## Supporting Information

S1 PRISMA Checklist PRISMA 2009 checklist. (DOC)

S1 Protocol The protocol registered in PROSPERO. (PDF)
