# Supplementary material for: Comparison of the Fertility Outcome of Salpingotomy and Salpingectomy in Women with Tubal Pregnancy: A Systematic Review and Meta-Analysis
Source: PLoS One. 2016 Mar 25;11(3):e0152343. doi: 10.1371/journal.pone.0152343 (PMC4807767; doi:10.1371/journal.pone.0152343)
Supplement: S1 Protocol — (PDF) [file pone.0152343.s002.pdf]

PROSPERO International prospective register of systematic reviews

Review title and timescale

1 Review title

Give the working title of the review. This must be in English. Ideally it should state succinctly the interventions or exposures being reviewed and the associated health or social problem being addressed in the review.

Conservative surgery versus salpingectomy in women with tubal pregnancy: a systematic review

2 Original language title

For reviews in languages other than English, this field should be used to enter the title in the language of the review. This will be displayed together with the English language title.

3 Anticipated or actual start date

Give the date when the systematic review commenced, or is expected to commence.

10/03/2015

4 Anticipated completion date

Give the date by which the review is expected to be completed.

31/12/2015

5 Stage of review at time of this submission

Indicate the stage of progress of the review by ticking the relevant boxes. Reviews that have progressed beyond the point of completing data extraction at the time of initial registration are not eligible for inclusion in PROSPERO. This field should be updated when any amendments are made to a published record.

The review has not yet started ×

Review stage	Started	Completed
Preliminary searches	Yes	No
Piloting of the study selection process	No	No
Formal screening of search results against eligibility criteria	No	No
Data extraction	No	No
Risk of bias (quality) assessment	No	No
Data analysis	No	No

Provide any other relevant information about the stage of the review here.

Funded proposal

Review team details

6 Named contact

The named contact acts as the guarantor for the accuracy of the information presented in the register record.

Miss Xiaolin

7 Named contact email

Enter the electronic mail address of the named contact.

cxlqhumc@126.com

8 Named contact address

Enter the full postal address for the named contact.

N0.24,Jinghua Road,Jianxi District,Luoyang City Henan Province

9 Named contact phone number

Enter the telephone number for the named contact, including international dialing code.

18637998243

10 Organisational affiliation of the review

Full title of the organisational affiliations for this review, and website address if available. This field may be completed as 'None' if the review is not affiliated to any organisation.

The First Affiliated Hospital of Henan University of Science and Technology

Website address:
http://yfy.haust.edu.cn/

11 Review team members and their organisational affiliations
Give the title, first name and last name of all members of the team working directly on the review. Give the organisational affiliations of each member of the review team.

Title	First name	Last name	Affiliation
Miss	Cheng	Xiaolin	The First Affiliated Hospital of Henan University of Science and Technology
Dr	Tian	Xiaoyu	The First Affiliated Hospital of Henan University of Science and Technology
Dr	Fan	Dongmei	The First Affiliated Hospital of Henan University of Science and Technology
Miss	Yan	Zhen	The First Affiliated Hospital of Henan University of Science and Technology
Miss	Jia	Mengmeng	The First Affiliated Hospital of Henan University of Science and Technology
Miss	Deng	Jie	The First Affiliated Hospital of Henan University of Science and Technology
Mr	Guo	Kelei	The First Affiliated Hospital of Henan University of Science and Technology,

12 Funding sources/sponsors
Give details of the individuals, organizations, groups or other legal entities who take responsibility for initiating, managing, sponsoring and/or financing the review. Any unique identification numbers assigned to the review by the individuals or bodies listed should be included.
none

13 Conflicts of interest
List any conditions that could lead to actual or perceived undue influence on judgements concerning the main topic investigated in the review.
Are there any actual or potential conflicts of interest?
None known

14 Collaborators
Give the name, affiliation and role of any individuals or organisations who are working on the review but who are not listed as review team members.

Title	First name	Last name	Organisation details

Review methods

15 Review question(s)
State the question(s) to be addressed / review objectives. Please complete a separate box for each question.
Will conservative surgery improve the rate of postoperative natural intrauterine fertility outcome compared with salpingectomy in the treatment of women with tubal pregnancy?

Is conservative surgery better than salpingectomy in terms of postoperative and complications in the treatment of women with tubal pregnancy?

16 Searches
Give details of the sources to be searched, and any restrictions (e.g. language or publication period). The full search strategy is not required, but may be supplied as a link or attachment.
The databases such as PubMed, MEDLINE, Cochrane Central Register of Controlled Trials (CENTRAL), EMBASE,The Cochrane Library, CNKI, and Wanfang database will be searched from their establishment to March 2015. The relevant magazines, the Conference Papers and the gray literature will also be searched. Any language

restriction or other limits will not be imposed on the searches.

17 URL to search strategy

If you have one, give the link to your search strategy here. Alternatively you can e-mail this to PROSPERO and we will store and link to it.

I give permission for this file to be made publicly available

Yes

18 Condition or domain being studied

Give a short description of the disease, condition or healthcare domain being studied. This could include health and wellbeing outcomes.

Tubal pregnancy is the most common type of ectopic pregnancy, and the incidence of tubal pregnancy has increased in recent years. Tubal ectopic pregnancy can be surgically treated by conservative surgery (salpingotomy) or salpingectomy. Usually, salpingotomy was widely adopted, because the tube is preserved, to a certain extent, it can increase the probability of intrauterine pregnancy. But Some studies show that after salpingotomy the damaged tube is likely to cause pelvic adhesions, hydrosalpin , this increases the risks of repeat ectopic pregnancy. Whether conservative surgery provides better fertility prospects than salpingectomy remains unclear.

19 Participants/population

Give summary criteria for the participants or populations being studied by the review. The preferred format includes details of both inclusion and exclusion criteria.

Women aged 18 years and older withß-HCG, an ultrasound scan or a laparoscopically confirmed tubal pregnancy receive conservative surgery or salpingectomy. Exclusion criteria: women with only one tube only,or with tubal occlusion or a hydrosalpinx, pregnant after in-vitro fertilisation (IVF) will also be excluded

20 Intervention(s), exposure(s)

Give full and clear descriptions of the nature of the interventions or the exposures to be reviewed

Conservative surgery: (usually salpingostomy) performed by laparoscopy or laparotomy, This surgical treatment conserves the affected tube. Salpingectomy: the ectopic conceptus is removed from the affected tube through a linear incision of the tube overlying the ectopic pregnancy whether the incision is surgically closed or not.

21 Comparator(s)/control

Where relevant, give details of the alternatives against which the main subject/topic of the review will be compared (e.g. another intervention or a non-exposed control group).

Tubal excision or salpingectomy performed by laparoscopy or laparotomy: The surgical removal of the tube affected by the ectopic pregnancy

22 Types of study to be included initially

Give details of the study designs to be included in the review. If there are no restrictions on the types of study design eligible for inclusion, this should be stated.

All randomised, quasi-randomised trials and cohort studies (prospective and retrospective) comparing conservative surgery and salpingectomy in the treatment of women with tubal pregnancy

23 Context

Give summary details of the setting and other relevant characteristics which help define the inclusion or exclusion criteria.

24 Primary outcome(s)

Give the most important outcomes.

The primary outcome was postoperative intrauterine pregnancy by natural conception.

Give information on timing and effect measures, as appropriate.

Including intrauterine pregnancy abortion and intrauterine pregnancy and delivery. To eliminate the artificial fertilization and embryo transfer assisted reproductive technology intrauterine pregnancy cases

25 Secondary outcomes

List any additional outcomes that will be addressed. If there are no secondary outcomes enter None.

Postoperative repeat tubal ectopic pregnancy, persistent ectopic pregnancy,infertility

Give information on timing and effect measures, as appropriate.

Persistent trophoblast was defined as the serum hCG concentrations still rising or plateauing two weeks after the surgical therapy. Repeat ectopic pregnancy was defined as any ectopic pregnancy or a persisting pregnancy of unknown location.

26 Data extraction, (selection and coding)

Give the procedure for selecting studies for the review and extracting data, including the number of researchers involved and how discrepancies will be resolved. List the data to be extracted.

27 Risk of bias (quality) assessment

State whether and how risk of bias will be assessed, how the quality of individual studies will be assessed, and whether and how this will influence the planned synthesis.

We will use the Cochrane 'Risk of bias' assessment tool to assess the risk of bias of all studies with a separate control group. Two author will independently assess risk of bias for each study, any disagreement will be resolved by consulting an independent person. Assessing risk of bias in randomised trials and quasi-randomised trials (1) Sequence generation (checking for possible selection bias) (2) Allocation concealment (checking for possible selection bias) (3) Similarity of baseline outcome measurements (checking for confounding, a potential consequence of selection bias) (4) Similarity of baseline characteristics (checking for confounding, a potential consequence of selection bias) (5) Incomplete outcome data (checking for possible attrition bias through withdrawals, dropouts and protocol deviations) (6) Blinding (checking for possible performance and detection bias) (7) Contamination (checking for possible performance bias) (8) Selective reporting bias (9) Other sources of bias

28 Strategy for data synthesis

Give the planned general approach to be used, for example whether the data to be used will be aggregate or at the level of individual participants, and whether a quantitative or narrative (descriptive) synthesis is planned. Where appropriate a brief outline of analytic approach should be given.

A meta-analysis will be conducted and a fixed-effect approach to the analysis will be used unless there was evidence of heterogeneity across studies. For non-randomised studies, where results have been adjusted to take account of possible confounding factors, we will use the generic inverse variance method in Review Manager 2014 to carry out any meta-analysis (If both adjusted and non-adjusted figures are provided we will carry out a sensitivity analysis using the unadjusted figures to examine any possible impact on the estimate of treatment effect). Results from randomised and non-randomised trials will not be combined in a meta-analysis.

29 Analysis of subgroups or subsets

Give any planned exploration of subgroups or subsets within the review. ‘None planned’ is a valid response if no subgroup analyses are planned.

Data were subgrouped if possible by the type of surgery and whether the incision is surgically closed or not in the following: 1.(laparoscopy approch) conservative surgery versus salpingectomy 2.(laparotomy approch) conservative surgery versus salpingectomy 3.conservative surgery (the incision is surgically closed) versus salpingectomy 4. conservative surgery (the incision not closed) versus salpingectomy

Review general information

30 Type of review

Select the type of review from the drop down list.

Intervention

31 Language

Select the language(s) in which the review is being written and will be made available, from the drop down list. Use the control key to select more than one language.

English, Chinese-Simplified

Will a summary/abstract be made available in English?

Yes

32 Country

Select the country in which the review is being carried out from the drop down list. For multi-national collaborations select all the countries involved. Use the control key to select more than one country.

China

33 Other registration details

Give the name of any organisation where the systematic review title or protocol is registered together with any unique

identification number assigned. If extracted data will be stored and made available through a repository such as the Systematic Review Data Repository (SRDR), details and a link should be included here.
none

34 Reference and/or URL for published protocol
Give the citation for the published protocol, if there is one.
Give the link to the published protocol, if there is one. This may be to an external site or to a protocol deposited with CRD in pdf format.

I give permission for this file to be made publicly available
Yes

35 Dissemination plans
Give brief details of plans for communicating essential messages from the review to the appropriate audiences.
Do you intend to publish the review on completion?
Yes

36 Keywords
Give words or phrases that best describe the review. (One word per box, create a new box for each term)
conservative surgery

salpingectomy

tubal pregnancy

37 Details of any existing review of the same topic by the same authors
Give details of earlier versions of the systematic review if an update of an existing review is being registered, including full bibliographic reference if possible.

38 Current review status
Review status should be updated when the review is completed and when it is published.
Ongoing

39 Any additional information
Provide any further information the review team consider relevant to the registration of the review.

40 Details of final report/publication(s)
This field should be left empty until details of the completed review are available.
Give the full citation for the final report or publication of the systematic review.
Give the URL where available.
